# Parental experiences of home phototherapy for neonatal hyperbilirubinemia

**DOI:** 10.1177/13674935221082404

**Published:** 2022-03-26

**Authors:** Miriam Pettersson, Mats Eriksson, Karin Blomberg

**Affiliations:** 1 Department of Paediatrics, Faculty of Medicine and Health, Örebro University, Örebro, Sweden; 2 Faculty of Medicine and Health, School of Health Sciences, 98837 Örebro University, Örebro, Sweden

**Keywords:** hyperbilirubinemia, neonatal, phototherapy, qualitative research

## Abstract

Newborns with hyperbilirubinemia have traditionally received phototherapy in hospital. Hospital stays for infants, however, may negatively affect parent–infant bonding and induce anxiety and feelings of powerlessness in mothers. This study examined parent’s experiences of providing phototherapy to their neonates at home instead. A descriptive qualitative study based on 15 interviews (8 mothers and 7 fathers) with parents of 8 children who had been randomised to home phototherapy was conducted during spring 2018 in Örebro county, Sweden. Inductive content analysis was used. The overall experience of home phototherapy was positive, and five categories were identified describing their experiences: continuing life at home, adjusting to having a newborn, feeling secure, experiencing parenthood and accessing information. The findings support the use of home phototherapy. Parents felt secure at home with their infants and emphasised the importance of clear information and round-the-clock access to hospital staff.

## Background

Neonatal hyperbilirubinemia is one of the most common adverse conditions in newborns (American Academy of Pediatrics Subcommittee on, 2004; Alkén et al., 2019). About 60% of term-born infants show clinical signs of hyperbilirubinemia, and about 2–3% of all such infants in Sweden receive phototherapy (Alkén et al., 2019). Signs of hyperbilirubinemia are yellow discolouration of the skin and sclera. If bilirubin levels are high, infants may also present with fatigue and feeding problems.

Neonatal hyperbilirubinemia is usually caused not by underlying disease, but by high levels of unconjugated bilirubin caused when red blood cells catabolise into bilirubin faster than the elimination rate (Rennie, 2012). The excess accumulates in body tissue and high levels are neurotoxic and need to be treated promptly to avoid brain damage (Alkén et al., 2019; American Academy of Pediatrics Subcommittee on, 2004; National Institute for Health and Care Excellence, 2016; Rennie, 2012).

Mothers have described having an infant with hyperbilirubinemia as physically and emotionally exhausting (Brethauer and Carey, 2010). They reported that hospitalisation of their baby robbed them of bonding time and they described a sense of loss of control and a negative impact on the rest of the family (Brethauer and Carey, 2010).

Mothers of infants with neonatal hyperbilirubinemia often felt guilty because they thought the condition was serious and they had somehow caused it (Hannon et al., 2001). They also worried that the treatment for hyperbilirubinemia would be unpleasant for their new baby (Hannon et al., 2001).

Phototherapy, the most common treatment for neonatal hyperbilirubinemia, has traditionally been provided during inpatient stays of one to 7 days (American Academy of Pediatrics Subcommittee on, 2004; National Institute for Health and Care Excellence, 2016). However, studies have reported that admitting a child to hospital can have a number of negative effects, including high levels of parental stress (Al Maghaireh et al., 2016; Balluffi et al., 2004; Grunberg et al., 2020; Roque et al., 2017).

Parent–child bonding is a process, which starts during pregnancy, and continues after birth (Wigert et al., 2006; Obeidat et al., 2009). The infant’s psychic and social development depends on early and continuous interaction and bonding with its parent to avoid negative effects later in life (Hedenbro and Rydelius, 2014; Puckering et al., 2014). Although one parent (often the mother) may stay with the child during hospital phototherapy in Sweden, this often means they are separated from the other parent and siblings, disrupting the family unit. This separation can be avoided by providing treatment at home. Previous studies have described parents’ experiences of their infant’s stay in hospital (Al Maghaireh et al., 2016; Balluffi et al., 2004; Grunberg et al., 2020; Roque et al., 2017), and the need for psychosocial support from the staff (Toivonen et al., 2020; Wigert et al., 2014), but only a few have described their experiences of treating their child for hyperbilirubinemia at home (Brethauer and Carey, 2010; Hannon et al., 2001).

The development of fibre-optic equipment has recently made home phototherapy an option for families. Performing this treatment at home could alleviate the stress parents and infants feel when they are separated during hospitalisation (Morgan et al., 2011). On the other hand, some parents might experience home phototherapy as a loss of contact with health care professionals that could lead to a feeling of insecurity. Thus, it is a need to further investigate experience from parents’ own perspective by chosen a qualitative design. To our knowledge, no other studies have addressed parents’ experience of home phototherapy for neonatal hyperbilirubinemia.

### Aim

To describe parents’ experiences of home phototherapy for neonatal hyperbilirubinemia.

## Methods

### Study design

This qualitative study was part of a randomised controlled multi-centre trial (focused on various aspects of home phototherapy for hyperbilirubinemia such as bonding, parental stress, impact on breastfeeding, and feasibility of home phototherapy. The quantitative measurements in the larger trial on the feasibility and safety of home phototherapy were published in January 2021 (Pettersson et al., 2021). Parents’ experience of receiving home phototherapy is relatively unknown, and it is therefore important to explore their perspectives to provide data to increase understanding of the intervention among health care professionals. A qualitative design allows to reach different perspectives on a phenomenon (Patton, 2015). We have followed the Consolidated Criteria for Reporting Qualitative Research checklist.

### Setting and sample

We conducted this study in the spring of 2018 in Örebro county, Sweden. Parents of infants randomised to receive home phototherapy were sampled consecutively, contacted by the first author, and asked to participate in interviews.

Inclusion criteria for the multi-centre home phototherapy study were infants with gestational weight >36, plasma bilirubin 300–400 µmol/l (17.5–23.4 mg/dl), and age >48 h at time of inclusion. Infants had to be otherwise healthy with no other medical conditions needing in-hospital treatment.

At time of inclusion, newborns were randomised to receive home phototherapy or standard in-hospital phototherapy. The home phototherapy group received treatment using the BiliSoft Phototherapy System 2.0 (GE Healthcare, Chicago, Il, USA). This fibre-optic equipment is easy to use and enables ongoing phototherapy with the infant in a cot, skin-to-skin with a parent, or feeding.

Before being sent home to initiate treatment, parents were given oral instructions on how to use the equipment and written information to contact the hospital at any time if questions or concerns should arise. During treatment, newborns and their parents returned to hospital for daily check-ups of the infant’s total serum bilirubin, weight, and general condition. Parents received standard instructions concerning feeding, not different from that given to all parents at the units (parents chose how to feed their newborn), but if an infant lost >10% of bodyweight, parents were advised on supplemental feeding such as additional formula.

New parent–infant dyads were recruited until no new information emerged in the interviews and data were considered saturated.

### Ethics

Ethical approval was obtained from (details omitted for peer review). All parents received oral and written information about the study, were informed of their right to discontinue participation at any time, and were assured this would not interfere with their infant’s care. Parents provided written consent before interviews were performed. The transcribed data were coded to protect participants’ identities and, in this paper, pseudonyms are used.

### Data collection

One author (MP) conducted individual face-to-face interviews at the parents’ homes (in line with parents own wishes) between January to April 2018, one to 2 weeks after home phototherapy ended. All parents were interviewed separately even if they both were at home. A semi-structured guide was used, focused on their experiences of home phototherapy. The interview-guide was pilot tested on one parent (by MP) and some wording was revised. All interviews started with short demographic questions such as parents’ ages and number of children in the family. Questions were then asked about parents’ experiences of providing home phototherapy, for example, ‘Please describe your experience of performing home phototherapy’ and ‘could you tell me about the information that you received before starting the phototherapy’. Follow-up questions encouraged them to elaborate on their responses, with examples if possible. All interviews were audio recorded and lasted 8–22 min. The pilot interview was included in final analysis due to its richness.

### Data analysis

Interviews were transcribed verbatim by a professional transcriber and subjected to inductive content analysis as described by (Elo and Kyngas, 2008). The first author (MP) read through the entire text several times and generated initial codes describing parents’ experiences of home phototherapy. Codes were placed on coding sheets and grouped into sub-categories based on their similarities and differences. The first author worked through the dataset several times and was open to adding new codes and refining the sub-categories. Categories were identified through abstraction. The first and last author (MP, KB) met to discuss the coding and provide additional insights. The whole research group reviewed the preliminary results to identify quotations illustrating the findings and increase trustworthiness of the study.

## Findings

Parents of eight infants (eight mothers and seven fathers) were interviewed for the study. Characteristics of the participants are presented in Table 1.

**Table 1.** Characteristics of participants.

	Number (%)	Median (Inter-quartile range)
Parents’ sex (Female/male)	8/7 (53/47)	
Age		27 (5)
Mothers		26.5 (4.5)
Fathers		28 (8)
Parity		1 (2)
Education (High school/college/unknown)	6/8/1 (40/53/7)	
Previous child hospitalised. (Yes/no)	2/13 (13/87)	
Hours of home phototherapy		17 (8)
Age of child at start of treatment (days)		3.8 (2.2)
Baby´s sex (Girl/boy)	5/3 (63/37)	

At 15 interviews no new experiences were described. We considered data saturation reached and conducted no further interviews.

Most parents were pleased to be able to give their child home phototherapy and to have been given an option. All said they would make the same choice again. Five categories described the parents’ experiences of giving their newborn infant home phototherapy: continuing life at home, adjusting to having a newborn, feeling secure, experiencing parenthood, and accessing information.

### Continuing life at home

Despite the changes that come with a newborn, parents reported that performing phototherapy at home allowed them to go back to their ordinary routines. They could go outside, have relatives to visit, and perform their everyday chores.

“Being at home you can continue your everyday life and you feel more relaxed.” (John)

Many parents discussed the perceived differences between staying at home and having their newborn admitted to hospital. Two parents compared the experience of home therapy with an earlier child’s hospitalisation and others talked about their own time in hospital for the birth or for other reasons. Hospitalisation gave them less freedom and made them feel more restricted. Several parents also said that if their infant had been admitted, they would have felt that their child was sick and it would have been more traumatic. Being able to provide phototherapy at home made the situation feel less dangerous.

“I think it feels more normal having phototherapy at home. If we had to stay at the hospital, it would have felt more severe.” (Emma)

Some parents, however, thought hospitalisation would let them get immediate answers to questions. A few said they may have felt more secure if their infant had stayed in hospital.

“Is this okay? Is it okay with a little light in the eyes or is it dangerous? Not being able to ask questions when you’re not secure.” (John)

“Not having someone to ask. You can’t just press the call button and ask… which stressed me a bit. And I didn´t dare to sleep at night, I wanted to sit and check that everything was okay… It was a long night.” (Anna)

Some parents said the phototherapy equipment affected their ability to continue with everyday life, but most felt it was practical and easy to use. This helped make the overall experience of home phototherapy positive. Parents felt no previous knowledge of the equipment was necessary and they felt secure using it at home. Some, however, mentioned issues they felt needed improving. For example, the newborns’ eye-shields were difficult to fasten. Because of this some parents did not use the phototherapy equipment during the night while they themselves were sleeping.

“So, it wasn´t difficult at all, you just had to plug it in.” (Rebecca)

“The glasses were difficult… they kept going up… they didn’t want to stay in place.” (Elisabeth)

### Adjusting to having a newborn

Parents felt that being able to perform phototherapy on their baby at home let them relax, recuperate, and adjust to having a newborn. Most described home phototherapy as allowing them to bond with their newborns, feel close to them, and hold them skin-to-skin during the treatment.

“There was a lot of bonding, he (the baby) lay skin-to-skin on my chest for almost 24 hours... it felt so good and we got really close…” (Alex)

“The baby slept really well. She lay on us, so we took turns having her skin-to-skin with the bilisoft blanket.” (Hannah)

When they were not holding their baby, it could be nearby in a cot or baby nest. Most parents felt their baby was comfortable receiving phototherapy.

Being able to feed their infant successfully was described as a vital part of adjusting to life with a newborn. Most said feeding was not complicated by providing phototherapy at home.

“Started breastfeeding her and there were no problems… I sort of tucked the bilisoft blanket around her.” (Hannah)

“The breastfeeding worked just fine… she started to gain weight and I felt really secure.” (Rebecca)

A few, however, felt that it was difficult to breastfeed while using phototherapy.

“It was a bit complicated to pick up the light blanket and feed her that way.” (Olivia)

“I had it in my arms, but it is a bit too clunky. It works, but the feeling was not the same either for her or me.” (Olivia)

Parents said they would have felt comfortable contacting the hospital for advice on feeding if they had concerns.

### Feeling secure

Most parents said the whole family felt safe and secure providing home phototherapy to their baby. One father even said that being at hospital would have made him feel more insecure than at home.

“Yes there´s a security in just being at home.” (John)

“I felt really safe with going home, otherwise I would have said so.” (Elisabeth)

“A downside (of being admitted to hospital) is that you feel less secure, you really just want to be at home…with the new-born.” (Daniel)

Several parents mentioned that the well-being of their newborn infant affected their sense of security. If their baby seemed to be doing well and was breastfeeding and gaining weight it helped them feel safe and secure using home phototherapy.

“I felt safe because the baby was eating and gaining weight. I felt safe that he was doing well” (Julia)

“The equipment was just really easy to use, there were no difficulties” (Hannah)

However, some described different areas of insecurity, such as whether the phototherapy equipment was efficient or not and whether the newborn infant would get too warm during phototherapy.

“It was good to be home, but it was also stressful because you didn´t really know if you did everything right.” (Anna)

“Since the baby was just a couple of days old you were extra tense and perhaps everything felt harder because it was so new…you just wanted her to breathe.” (Rebecca)

Some parents said they felt unsure about handling and taking care of their newborn because it was so young and it was their first child.

One couple expressed feeling very insecure about receiving inadequate information before going home to start phototherapy. They felt stressed about whether they had handled the equipment properly, and the mother felt especially insecure.

“It was quite stressing to be at home, because you didn’t know if you handled it right, will it give any result? Should it be like this? Yes, I felt I had many questions.” (Anna)

### Experiencing parenthood

Most parents talked about how their roles affected their experience of home phototherapy. Not being home alone with their newborn and being responsible for the home treatment were crucial issues.

“We became a family that helped each other.” (Julia)

“The mother and father shared the responsibility for the baby and for the treatment.” (David)

“I wouldn´t have wanted to be alone doing the phototherapy. It was almost a requirement that you needed two to cope and to make sure the baby got a lot of phototherapy.” (Julia)

Parents generally felt it necessary and desirable to have two people at home during phototherapy, as this made them feel more confident about handling the treatment. They took turns monitoring the newborn, providing its treatment, and looking after it during skin-to-skin contact and while it was in its cot or baby nest. Several mentioned that it was good to have the family home during phototherapy because the treatment became part of everyday life.

“During the nights there were long periods of time when I had her skin-to skin, on my chest.” (John)

“You felt important to your child… there were a lot of people saying that you really don´t know what role you’re supposed to have when you come home with the new-born… but it was so natural, you could actually contribute to the whole thing.” (Alex)

A few men talked about their experiences of fatherhood and felt that they had a natural role to play during phototherapy. They felt they participated in the care of their newborn, providing phototherapy and spending a lot of time skin-to-skin with the baby during treatment. One father mentioned how important this was and how it helped him to feel close to his newborn. Other fathers took on a more indirect role, such as looking after siblings of the newborn and managing everyday practicalities.

“It felt like… there was time to recuperate… you could just be yourself.” (Julia)

“I was the one that mainly took care of it [the newborn and the phototherapy] and since we have an older child, my husband took care of him… also since I was breastfeeding the baby.” (Alice)

Some mothers felt they had main responsibility for taking care of the newborn and its phototherapy and saw this as part of the maternal role. One mother, however, said she was pleased that her partner could get involved in the treatment as it gave her time to recover from giving birth.

### Accessing information

All parents discussed the information they were given at hospital before they were discharged with their infant and the phototherapy equipment. Information about the treatment affected their experiences of home phototherapy.

“It felt like I received good information and they showed me how to use the equipment… there were really no difficulties…” (Alice)

“When we unpacked the equipment and started (the phototherapy) … the information given beforehand corresponded well to how it actually turned out… everything just worked.” (Alex)

“Well, it was the information that we got…I felt it was thorough and good… so I felt secure.” (Eric)

Most understood why phototherapy was needed and felt they had been given thorough and relevant information about how to perform it at home. Some said when they lacked information, they searched the Internet or asked relatives or friends. Most said they would have liked more information about how long their newborn infant’s skin would remain jaundiced after treatment and whether light from the equipment could damage its eyes. Some found it hard to remember verbal information and would have liked to have it in writing as well.

“Having to visit the hospital the day after to check the results… well it felt super good and super safe… there was security in having a revisit almost immediately.” (David)

Most parents felt more secure knowing they could ask hospital staff if they had any queries or concerns. The infants’ daily hospital check-ups added to their sense of security.

The few parents who did not realise they could call the hospital with questions felt less secure at home than others who felt better informed and had fewer questions.

## Discussion

The aim of this study was to describe parent´s experiences of home phototherapy. Findings show their overall experiences were positive, although some had questions and concerns. All interviewees said that if they had to choose again between providing phototherapy at home or staying in hospital with the infant, they would choose home treatment.

Research has shown that parents have higher levels of stress and anxiety during and after their children’s hospitalisations (Obeidat et al., 2009). Most such studies concern parents with children in intensive care, either paediatric (Balluffi et al., 2004) or neonatal (Al Maghaireh et al., 2016; Roque et al., 2017), but similar findings have been reported for more general paediatric or neonatal care (Franck et al., 2015; Wray et al., 2011). These studies support the idea of providing care and treatment at home when feasible to avoid stressing families with unnecessary separation and an unfamiliar hospital environment. Some parents in our study felt stressed by having primary responsibility for their child’s home treatment, but all reported the overall experience as positive and none would have preferred hospital treatment.

Separation early in life can negatively impact the vital parent–child bonding process (Hedenbro and Rydelius, 2014; Puckering et al., 2014), leading to increased stress and negative effects on the baby (Morgan et al., 2011). Such early separation can damage the attachment process and have long-lasting effects on the child’s emotional programming (Curley and Champagne, 2016; Flacking et al., 2012; Lupien et al., 2009; Sullivan et al., 2011). Being able to leave hospital for home phototherapy decreases the negative impact of family separation as parents can continue to be the infants’ main caregivers. Parents in our study felt that home treatment gave them sufficient time to bond with their newborn infant, making it easier to adapt to their new arrival. They felt close to their baby and that the skin-to-skin contact they experienced during long periods of phototherapy helped.

Other findings included how parents experienced parenthood and described their roles during home phototherapy, especially their feelings that it was necessary for both to stay home during treatment and to feel safe providing that aspect of their newborn’s care. It should be noted that all participants were in couples. In future, clinicians should consider the support single parents would need were they offered the alternative of home treatment. Having a friend or relative to assist them could contribute to their overall performance and satisfaction.

Previous research has described how mothers felt about having a newborn with neonatal jaundice undergo phototherapy (Brethauer and Carey, 2010; Hannon et al., 2001). To the best of our knowledge, our study is the first in which fathers also describe their experiences of having an infant with that condition. Some fathers in our study described playing a rather traditional role and including handling practical issues while the mother cared for the infant. Others described how taking care of their newborn during home phototherapy reinforced their natural caregiver role, especially during long periods of skin-to-skin contact during therapy. This is in line with a previous study in which fathers reported that skin-to-skin care made them feel more included in the care of their newborn (Olsson et al., 2017).

A possible negative effect of home therapy could be parents feeling more insecure outside the immediately available care in hospital. Most parents in our study, however, did not express such feelings. In general, being at home gave them a sense of security. This feeling was supported by the ease of using the equipment and the well-being of the baby.

Findings also showed the importance of providing parents with relevant and structured information before they go home to treat their newborn infant on their own. Most parents felt the information provided was adequate, but those who did not feel well informed were clearly more insecure. This finding is supported by other studies showing that adequate information reassured parents whose babies were ill and undergoing treatment (Russell et al., 2014) and parents of full-term infants after their discharge from hospital (Henshaw et al., 2018; Wiklund et al., 2018). Two easy ways to enhance information for parents is to provide it both verbally and in writing and to ensure parents understand they can contact the hospital with questions at any time.

In our study, parents were clear that regular check-ups at hospital during their infants’ treatment reassured them and made them feel secure. This security could be enhanced by clarifying specific aspects of the treatment. Some parents were afraid to sleep or to perform therapy at night because they were not confident the baby’s eye-shield would stay on. For parents to feel safe and deliver the therapy effectively, it is important they be told how important it is to continue phototherapy through the night and how to fasten the eye-shields properly.

## Limitations

The selection of the parents in our study was both a strength and limitation. By using consecutive sampling, we recruited participants with geographical and educational differences. However, we were unable to include parents who did not speak Swedish. Future studies on home phototherapy should include families with greater ethnic diversity to determine whether treatment is also feasible for them. Duration of the interviews could be seen as short, but the phenomenon being studied is concrete and the intervention relatively limited in time (17 h on average). Overall, collected data includes a range of variations responding to the aim of the study.

## Implications for practice

To change a practice to shift much of the responsibility for its implementation to parents, it is important to investigate and describe parents’ experiences. Previous studies have stated the feasibility and safety of home phototherapy (Pettersson et al., 2021). This study, which goes further by exploring parents’ experiences, supports implementation and routine use of home phototherapy.

## Conclusion

All parents in our study described how being able to treat their infant at home helped them adapt to everyday life and enhanced their positive experience of parenthood. Overall, they felt safe handling the treatment at home, though many found that it was necessary that both parents stayed home during treatment. Fathers in particular described playing a natural role in providing their infant’s therapy and felt that caring for their infant this way was a positive part of the bonding process. Factors enhancing parent´s sense of security were good quality information before their infant was discharged and being able to contact hospital staff round-the-clock with any questions or concerns. In those cases, when parents did not feel well informed, they felt more insecure. Our findings also highlighted that regular check-ups were essential during treatment.
